# Optimization of procedural sedation and analgesia during atrial fibrillation ablation

**DOI:** 10.1097/ACO.0000000000001263

**Published:** 2023-03-23

**Authors:** Marloes C. Homberg, Esther A.C. Bouman, Bert A.J. Joosten

**Affiliations:** Marloes Homberg, Department of Anesthesiology and Pain Medicine, Maastricht University Medical Center+, Maastricht, The Netherlands

**Keywords:** atrial fibrillation ablation, deep, minimal, moderate, sedation

## Abstract

This mini-review is aimed to provide an overview and discuss procedural sedation and analgesia for atrial fibrillation (AF) ablation with focus at qualification of staff, patient evaluation, monitoring, medication and postprocedural care.

**Recent findings:**

Sleep-disordered breathing is highly prevalent in patients with AF. Impact of often used STOP-BANG questionnaire to detect sleep-disordered breathing in AF patients is limited due to its restricted validity. Dexmedetomidine is a commonly used drug in sedation, but is shown not to be superior to propofol in sedation during AF-ablation. Alternatively use of remimazolam has characteristics that makes it a promising drug for minimal to moderate sedation for AF-ablation. High flow nasal oxygen (HFNO) has shown to reduce the risk of desaturation in adults receiving procedural sedation and analgesia.

**Summary:**

An optimal sedation strategy during AF ablation should be based on AF patient characteristics, the level of sedation needed, the procedure (duration and type of ablation) and the education and experience of the sedation provider. Patient evaluation and post procedural care are part of sedation care. More personalized care based on use of various sedation strategies and types of drugs as related to the type of AF-ablation is the way to further optimize care.

## INTRODUCTION

Catheter ablation for atrial fibrillation (AF) has emerged as an important rhythm-control strategy and is by far the most common cardiac ablation procedure performed worldwide [1]. Radiofrequency ablation (RFA) with 3D electro-anatomical mapping to guide the catheter and cryoablation are techniques for AF-ablation [1]. The best modality of anesthesia with catheter ablation for AF is still a matter of debate [2^▪^]. Although general anesthesia is the standard in the majority of clinical centers, procedural sedation and/or local anesthesia is a good alternative and used in those clinical centers where general anesthesia is not easily available [2^▪^]. Sedation is not inferior to general anesthesia in terms of ablation time and freedom from atrial arrhythmia at 1 year and accompanied by reduced resource utilization and therefore costs [3]. 

**Box 1:**
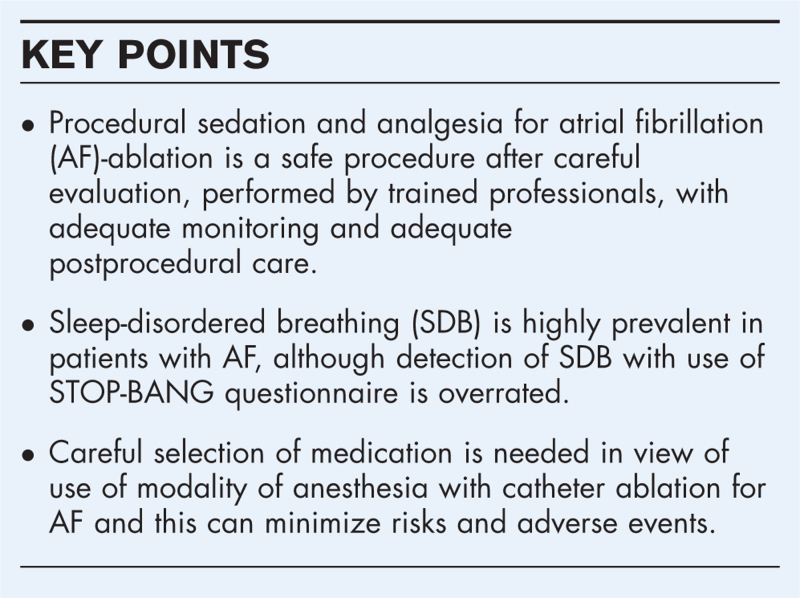
no caption available

Procedural sedation and analgesia (PSA) involves the use of hypnotic and/or analgesic medications to enable diagnostic or therapeutic procedures effectively, whilst the patient is closely monitored for potential adverse effects [4]. Depth of sedation is graded into: minimal sedation, moderate sedation, deep sedation and general anesthesia (Table 1) based on the recommendations of the American Society of Anesthesiologists (ASA) [5]. Although the wording conscious sedation is commonly used, this term is misleading and should not be used as it is contradictory as effective sedation decreases consciousness [4]. For AF cryoablation minimal or moderate sedation have been shown to be efficient in the majority of patients [6]. For RFA with 3D electro-anatomical mapping deep sedation or general anesthesia is mandatory as the success of the procedure depends on minimal patient movement [6].

**Table 1:** Levels of sedation as suggested by the American Society of Anesthesiologists [4]

	Level 1 Minimal	Level 2 Moderate	Level 3 Deep	Level 4 General anaesthesia
Responsiveness	Normal responses to verbal stimuli. Cognitive function and coordination may be impaired	Purposeful responses to verbal/tactile stimuli and verbal commands	Not easily rousable. Purposeful responses to repeated verbal/tactile or painful stimuli.	Not rousable. No response to painful stimulation
Airway	Normal	Airway is patent	Ability to maintain a patent airway may be impaired. Airway support may be required	Ability to maintain a patent airway Airway support is required.
Ventilation	Normal	Spontaneous ventilation is adequate	Ability to maintain spontaneous ventilation may be impaired. Respiratory support may be required	Ability to maintain spontaneous ventilation is impaired Respiratory support and positive pressure ventilation is required.
Haemodynamic function	Normal	Unaffected	Usually unaffected	May be impaired

Previously published table. European Society of Anaesthesiology and European Board of Anaesthesiology guidelines for procedural sedation and analgesia in adults. J Hinkelbein Jochen. Eur J Anaesthesiol 2018;35:6–24. https://cdn-links.lww.com/permalink/eja/a/eja_2017_06_28_lamperti_eja-d-17-00270_sdc1.doc.

In clinical practice, it is not always possible to predict how an individual patient will respond to sedative agents, and for instance moderate sedation can unintentionally lead to deep sedation [5]. Providers are responsible for resuming a well tolerated level of sedation while ensuring patients safety. Complications during AF-ablation are sedation and/or procedure related. The complications related to sedation are mostly a consequence of the effects and adverse effects of the sedation agents. The most common drug-related side-effects are apnea, airway obstruction, hypoventilation and hypotension. A recent study on drug-related complications in a cohort of patients undergoing deep sedation for AF-ablation showed that out of 3211 patients 1 patient (0.03%) desired endotracheal intubation (eIT) and 47 patients (1,5%) required noninvasive ventilation (NIV) [7^▪▪^]. Those patients which needed eIT and/or NIV experienced an increased duration of sedation procedures, an increased CHA2DS2–VASC score and were characterized by a higher body mass index (BMI) [7^▪▪^]. The CHA2DS2–VASC score is widely used to estimate cardio-embolic risk in patients with atrial fibrillation [8]. In this CHA2DS2–VASC score one point is related to each of the following individual parameters: congestive heart failure, hypertension, age between 65 and 74 years, diabetes mellitus, vascular disease (myocardial infarction/peripheral arterial disease) and female gender (one point for each parameter) whereas two points are related to previous Stroke/Transient ischemic attack and to age ≥75 years [8].

The rate of procedure related complications during deep sedation for AF-ablation is approximately 2.9% and has decreased over the years [9]. It needs to be stressed that although sedation and procedure related complications are rare, complications like cardiac tamponade, stroke, pulmonary vein stenosis, vascular access related complications (e.g. bleeding, hematoma, femoral pseudoaneurysm formation) and pneumothorax occur rapidly, are often very serious and may be life-threatening [9].

As sedation is dynamic and the course of patients may vary considerably, we will discuss the following topics in more detail: qualification of staff involved in PSA with AF ablation, patient selection, and peri-operative care during AF ablation.

## QUALIFICATION OF STAFF INVOLVED IN PROCEDURAL SEDATION AND ANALGESIA FOR ATRIAL FIBRILLATION ABLATION

The growing demand for PSA for AF ablation cannot be fully covered and performed by anesthesiologists in a number of countries. It is clinical reality that not only anesthesiologists but also other healthcare practitioners like cardiologists, nurse practitioners, physician assistants, nurses and sedation practitioners (SPs) provide PSA for AF-ablation. In view of the complexity of the procedure and/or treatment it is mandatory that sedation is performed by a sedation provider with adequate qualification and full focus on PSA.

Fundamental knowledge of sedative agents, analgesics, cardio-active and antagonist drugs is mandatory and are standards to maintain [10]. The most common risk associated with PSA is respiratory compromise including hypoventilation and apnea potentially resulting in hypoxemia [10]. The sedation provider is expected to have airway management skills including mask ventilation, use of supraglottic airway devices and the use of advanced airway devices like laryngeal mask airway (LMA) and endotracheal intubation [10]. As there is a need for formal training programs, some professional national and international anesthesiology-societies developed guidelines and a curriculum like the ASA's Safe Sedation Training. The latter is an educational program designed to guide moderate and deep sedation for nonanesthesiologist and healthcare providers [10]. For example, in the Netherlands, SPs are certified anesthesia nurses trained at the anesthesia department, in line with the European Society of Anesthesiology and Intensive Care (ESAIC) guidelines on PSA of adults [4]. Nevertheless, moderate to deep sedation by SPs is often performed under indirect supervision of an anesthesiologist [2^▪^].

It has been demonstrated that this setting of trained SPs as in the Netherlands is safe as sedation and sedation-related events have been reported to be managed effectively [11]. In case of complex sedation or high-risk procedures, the SPs can request for direct supervision of an anesthesiologist. Legal requirements for PSA differ all over the world [2^▪^,^7^^▪▪^]. For example, the United States Food and Drug Administration (FDA) only approves the use of propofol to perform deep sedation by trained sedation providers due to its risk profile [12]. On the other hand in countries like France, Spain or Italy, it is not permitted to perform deep sedation without the presence of an anesthesiologist [2^▪^].

Qualifications of staff involved in PSA with AF ablation under moderate to deep sedation are of utmost importance for optimal care and need to be well described for the sedation provider in each institution. Training and (in)direct supervision of a sedation provider by an anesthesiologists is closely related to the complexity of the case and/or procedure. Guidelines and practice recommendations as published by ESAIC and/or ASA should be implemented in local practice and provide essential basic information (Table 2).

**Table 2:** Summary of suggested training expectations for sedation providers of moderate and deep sedation [10]

Training expectation	Moderate sedation	Deep sedation
Anesthetic provider	Anesthesia trained professionals (anesthesiologist, CRNA, or AA), nonanesthesiologist providers, and supervised sedation practioners (registered nurses and physician assistance)	Anesthesia trained professionals (anesthesiologist, CRNA, or AA), nonanesthesiologist providers (doctor of medicine or osteopathy, qualified dentist, oral surgeon, or podiatrist)
Approved sedatives	Opioids, benzodiazepines, and dexmedetomidine	Opioids, benzodiazepines, dexmedetomidine, propofol, and ketamine
Emergent life support	ACLS & mastery of moderate sedation and recognition of deep sedation	ACLS & mastery of deep sedation and recognition of general anesthesia
Airway management skills	Face mask and positive pressure ventilation	Bag mask ventilation, oro- and nasopharyngeal airway, laryngoscopy and ET intubation
Preprocedural evaluation	History and physical	History and physical Formal perioperative evaluation
Monitoring and Documentation	Pulse oximetry, BP, EKG, HR, depth of sedation, capnography	Pulse oximetry, BP, EKG, HR, depth of sedation, capnography

AA, anesthesiologist assistant; ACLS, advanced cardiac life support; BP, blood pressure; CRNA, certified registered nurse anesthetists; EKG, electrocardiogram; ET, endotracheal tube; HR, heart rate. Previously published table. Moderate and deep sedation training and pharmacology for nonanesthesiologists: recommendations for effective practice. T.T. Tran Thi. Curr Opin Anesthesiol 2019;32:457–463.

## PATIENT EVALUATION

Preprocedural patient evaluation contributes to the likelihood of satisfactory sedation and decreases the likelihood of adverse outcomes for both moderate and deep sedation [10,13]. The following recommendations are widely accepted: patient evaluation for elective PSA in advance to allow optimal patient preparation, perform a standard preanesthesia assessment, reviewing previous medical records and interview the patient, including focused physical examination, and review available laboratory test results [13]. Based on the outcome of the interview and the physical assessment additional examination might be needed. For example, kidney function is necessary preprocedural for AF-ablation, medication dosage reduction can be indicated if kidney function is impaired and further reduction caused by contrast-induced nephropathy avoided.

Furthermore, patients receiving sedation for AF-ablation usually require preprocedural cardiac echocardiography and evaluation for obstructive sleep apnea syndrome (OSAS). As AF-ablation is an elective procedure there is time to perform a preprocedural evaluation in advance and to optimize the condition of the patient if indicated.

Patients with a significant comorbidity (like those with suspected high risk of either OSAS, or severe cardiovascular diseases, or morbid obesity, or chronic renal failure, or chronic hepatic disease as well as elderly or patients with an ASA Physical Status III and IV [4,13]) require evaluation and management of procedural sedation and analgesia by an anesthesiology professionals like nurse anesthetists, screeners, residents or anesthesiologists [4]. In case of preexisting renal failure or/and chronic hepatic disease adverse events can be reduced by careful selection of medication, adjustment of dose and careful titration for example in patients with reduced clearance. A BMI >30 kg/m^2^ has been shown to be an independent predictor for severe persistent hypoxemia during sedation for AF-ablation [7^▪▪^]. OSAS is the most common subtype of sleep-disordered breathing (SDB) and is highly prevalent in AF-patients [14^▪^]. Detection of SDB is by use of the STOP-BANG questionnaire [15]. Nevertheless impact of this questionnaire is limited due to its restricted validity to detect SDB in AF patients [16,17]. Most patients with AF do not report daytime sleepiness, a typical SDB- related symptom [14^▪^]. Diagnostics for SDB should not only be performed to reduce adverse sedation events but SDB is also a predictor of a twofold higher risk of AF recurrence after ablation [18].

In conclusion: Careful patient evaluation contributes to patient safety as it signals the risk factors for sedation related adverse events. Severe OSAS and morbid obesity are relative contra-indications for moderate to deep sedation during AF-ablation; then general anesthesia is a safe alternative. As the threshold of parameters resulting in the need for general anesthesia do not exist this is a target for future research.

## PERI-PROCEDURAL CARE DURING AF ABLATION

Peri-procedural sedation care during AF ablation can be subdivided into 3 areas of interest:

(1) Monitoring,
(2) Medication,
(3) Postprocedure care.

## MONITORING

Standard monitoring of patients undergoing PSA includes noninvasive blood pressure (NIBP), pulse oximetry, ECG and end expiratory capnography (ExCO_2_). Some authors recommend the use of depth of sedation monitoring (e.g. processed EEG) [4,13]. During moderate or deep sedation continuous evaluation of ventilation using ExCO2 is mandatory. End expiratory CO_2_ monitoring is able to detect apnea earlier as compared to use of pulse oximetry [4]. The use of ExCO_2_ has shown to reduce the incidence of hypoxic events by early detection of apnea [19]. During ExCO_2_ monitoring waveform abnormalities can be observed and the measurement of ExCO_2_ can be unreliable. If it is technically not possible to measure reliably ExCO_2_, for example using high flow nasal oxygen, peri-procedural transcutaneous carbon dioxide level (TcCO_2_) is an noninvasive option to monitor CO_2_[19]. TcCO_2_ measurement is an alternative used during PSA as a replacement for laboratory measurement of blood CO_2_ levels [19,20^▪^]. However invasive arterial blood pressure monitoring can be indicated in selected cases. Foerscher recently observed and reported that arterial blood pressure monitoring is indicated for blood pressure control and/or arterial blood gas analysis in 27.8% of the population in a study in patients undergoing left arterial ablation [7^▪▪^]. The results of this study also showed also a significant blood pressure drop (mean arterial pressure < 60 mmHg) in 12.3% of the cases.

The level of sedation can be rated by the Ramsey Sedation Scale (RSS) or Modified Observer's Assessment of Alertness/Sedation (MOAA/S) (Table 3). Processed EEG like bispectral index (BIS) monitoring is used to measure the level of sedation. It has been demonstrated that EEG based titration of hypnotic agents leads to a stable level of sedation [4]. This stable situation consequently results in increased patient as well as staff satisfaction and reduces the duration of the procedure resulting in enhanced productivity. The latter seems important giving the long waiting times for patients planned for elective ablation.

**Table 3:** Sedation scales commonly used in PSA

	Ramsey Sedation Scale (RSS)	MOAA/S Scale
0		Does no trespond to painful trapezius squeeze
1	Patient is anxious and agitated or restless, or both	Responds only after painful trapezius squeeze
2	Patient is cooperative, oriented and tranquil	Responds only after mild prodding or shaking
3	Patient responds to commands only	Responds only after name is called loudly and/or repeatedly
4	Patient exhibits brisk response to light glabellar tap or loud auditory stimulus	Lethargic response to name spoken in normal tone
5	Patient exhibits a sluggish response to light glabellar tap or loud auditory stimulus	Responds readily to name spoken in normal tone
6	Patient exhibits no response	

PSA, procedural sedation and analgesia.

Improvements in monitoring of patients undergoing AF-ablation are still possible using noninvasive methods like TcCO_2_ for monitoring ventilation. These noninvasive methods allow early detection of hypo-ventilation. Correct adjustment of the depth of sedation does easily solve these ventilation problems and thus prevents the occurrence of any sedation related complications.

## MEDICATION

### Hypnotic agents

Midazolam and/or fentanyl is commonly used with minimal to moderate sedation [2^▪^,^10^]. For deep sedation propofol [continuous or target controlled infusion (TCI)] is most often used as sedative, alone or in combination with midazolam [2^▪^,^10^]. In a recent survey the current status and evolution of the use of sedation strategies for AF-ablation was presented and there it was indicated that over the last decade propofol is more frequently used for deep sedation (57.2%) as compared to midazolam (48.1%) [2^▪^]. In the same review, it is documented that with minimal sedation midazolam is preferred to be used (in 58.6%) over propofol (19.2%) [2^▪^].

Other commonly used drugs in sedation of patients with AF-ablation are dexmedetomindine and ketamine. Dexmedetomidine (dexmed) is an alpha2-adrenoreceptor agonist with sedating and analgesic effects without respiratory depression [21]. Decreased heart rate via sinus and AV node depression and hypotension caused by decreased vascular resistance occur with the use of dexmed [21]. In patients with underlying heart disease dexmed may cause left ventricular dysfunction [21]. In a recent study, Servatius *et al.*[22^▪▪^] compared the effects of propofol vs. dexmedetomidine in 160 patients undergoing radiofrequency catheter ablation. Dexmed was shown not to be superior to propofol in this randomized controlled study. Hypercapnia occurred less frequent with dexmed, but with propofol the sedation strategy was shown to be efficient in all patients and patient satisfaction was significantly higher [22^▪▪^].

Ketamine is a phencyclidine-derived anesthetic which can be used in deep sedation [10]. It is an unique anesthetic because it can be used as a sedative and as an analgesic [21]. Because of its hypertensive side effects attention is needed in particular in patients with hypertension and/or coronary artery disease. With use of ketamine not only respiratory drive but also heart rate and airway reflexes are generally (better) preserved as compared to that noted after use of other drugs/sedatives [10]. Ketamine induced side-affects like dissociation and hallucinations can cause patient movement, psychotomimetic emergence and hyper-salivation and they do relatively often occur [10]. Sub-therapeutic doses of ketamine provide analgesia in AF-patients who are already sedated with propofol for example. A derivative of ketamine, esketamine might become an interesting option providing more potent sedative and analgesic effects and less psychotropic side effects [23].

Remimazolam is a more recently used drug that undergoes rapid metabolism and degradation due to abundant presence of plasma and tissue esterases. The safety profile of remimazolam is comparable to midazolam although sedation depth is reached much faster [24]. With respect to remimazolam, all present studies and randomized controlled trials (RCTs) are performed during sedation for endoscopies and therefore the results cannot be generalized to sedation for AF-ablation. In a meta-analysis of five RCTs with 1248 participants, Jhuang *et al.*[25] investigated the effectiveness and adverse events (AEs) of remimazolam during procedural sedation. Compared with the use of midazolam, the utilization of remimazolam resulted in an increase procedure success rate, a reduction in the application of rescue medication, a decrease in time to recovery and a better cognitive recovery of Hopkins Verbal Learning Test-Revised [25]. Theoretically, the use of remimazolam for mild to moderate sedation in AF-ablation can be beneficial. Hence the use of remimazolam in AF ablation and mild to moderate sedation is an important target for future research.

### Analgesic drugs

Most commonly, hypnotic agents are combined with opioids for optimal pain relief. The opioid remifentanil is a fast acting drug which can be titrated accurately by TCI and this drug is characterized by a fast wash out in minutes after ending infusion (T1/2 el 12–30 min) whereas the duration of action of opioid fentanyl is 30–60 min (T1/2 el 6–8 h) [10]. Remifentanil is fastly hydrolyzed and degraded and cleared due to abundant presence of plasma and tissue esterases [24]. Fentanyl is metabolized via CYP3A4 pathways and is renally cleared and this makes that the dosage should be adjusted in case of liver or renal failure. Information on the preference of the use of opioids for moderate and deep sedation is rather limited and can at present not exactly be determined as in a recent survey fentanyl and remifentanil were combined and analyzed as one group [2^▪^].

Combination and use of drugs via one bolus of midazolam, continuous infusion of propofol and boluses fentanyl is often used for deep sedation of patients during AF-ablation [7^▪▪^]. The combined use of TCI of propofol and remifentanil is expected to increase among anesthesia sedation providers as TCI facilitates precise control over the level of sedation [7^▪▪^].

## POSTPROCEDURE CARE

The ESAIC guidelines on procedural sedation recommend that patients after PSA must be monitored in a recovery room for at least 30 minutes [4]. Postsedation monitoring (with at least NIBP, ECG and pulse oximetry) is essential to supplement continuous visual observation by an experienced trained nurse. Patients may deteriorate considerably after PSA and AF-ablation, mostly patients with a significant disease burden or with complications of the AF-ablation. Post procedure management includes pain management, HD optimization and recognizing and treatment of postprocedure complications. Most major complications were reported to occur intraprocedurally or within 6 h after the procedure [26]. Congestive heart failure and transient ischemic attack were the most common major complications [26].

## OPTIMIZING CARE

Literature concerning patients with OSAS undergoing moderate and deep sedation is lacking, also for AF-ablation procedures. SBD-screening prior to PSA can result in the reduction of respiratory complications during sedation [27]. In case of severe OSAS general anesthesia is an alternative for deep sedation. As the threshold of parameters resulting in the need for general anesthesia do not exist this is a target for future research. Further studies are needed to determine if moderate and deep sedation can safely be performed in SBD patients. TcCO_2_ monitoring should be considered in patients with risk for hypercapnia and acidosis like those with obesity, chronic obstructive pulmonary disease and SBD (all common in AF-patients). Supporting spontaneous ventilation during sedation with high flow nasal oxygen (HFNO) or NIV may result in optimization of ventilation. HFNO has been shown to reduce the risk of desaturation in adults receiving PSA [28^▪^]. Further studies are needed to show if HFNO/NIV during deep sedation indeed reduces hypercapnia and this should be linked to the identification of those patients who will benefit from HFNO/NIV. Titration of propofol and remifentanil with TCI may facilitate precise control over the level of sedation. TCI is available in many countries but is not yet approved in the United States by Food and Drug Administration (FDA) [24].

## CONCLUSION

An optimal sedation strategy during AF ablation should be based on AF patient characteristics, the level of sedation needed, the procedure (duration and type of ablation) and the education and experience of the sedation provider. Careful medication selection can minimize risks and adverse events. Patient evaluation and post procedural care are part of sedation care. New (combinations of) drugs, noninvasive monitoring like TcCO_2_ and optimizing ventilation during sedation may further optimize sedation care during AF-ablation. More personalized care based on use of various sedation strategies and types of drugs as related to the type of AF-ablation is the way to further optimize care. Guidelines and practice recommendations as published by ESAIC and/or ASA should be implemented in local practice. Training and (in)direct supervision of a sedation provider by an anesthesiologists is closely related to the complexity of the case and/or procedure.

## Acknowledgements

*We thank Prof. Dr Wolfgang Buhre (Department of Anesthesiology at Utrecht University Medical Center) and Dr Dominik Linz (Department of cardiology at Maastricht University Medical Center+) for their expertise and assistance.*

### Financial support and sponsorship

*This work was supported by the Department of Anesthesiology, Maastricht University Medical Centre, Maastricht, The Netherlands.*

### Conflicts of interest

*There are no conflicts of interest.*

## REFERENCES AND RECOMMENDED READING

Papers of particular interest, published within the annual period of review, have been highlighted as:

▪ of special interest
▪▪ of outstanding interest
